# Designing systems for the care we need: A transformation journey in Southwestern Ontario

**DOI:** 10.1177/08404704231178456

**Published:** 2023-06-15

**Authors:** Shannon L. Sibbald, Jacobi Elliott, Alexander Smith, Mulugeta B. Chala, Nancy Dool Kontio, Amber Alpaugh-Bishop, Sarah Jarmain, Atharv Joshi, Mike McMahon, Matthew Meyer

**Affiliations:** 1 6221 Western University, London, Ontario, Canada.; 2 St. Joseph's Health Care London, London, Ontario, Canada.; 3 10033 London Health Sciences Centre, London, Ontario, Canada.; 4 274071 University of Toronto, Toronto, Ontario, Canada.; 5 Middlesex-London Ontario Health Team, London, Ontario, Canada.; 6 Thames Valley Family Health Team, London, Ontario, Canada.

## Abstract

Primary care is considered the foundation of any health system. In Ontario, Canada Bills 41 and 74 introduced in 2016 and 2019, respectively, aimed to move towards a primary care-focused and sustainable integrated care approach designed around the needs of local populations. These bills collectively set the stage for integrated care and population health management in Ontario, with Ontario Health Teams (OHTs) introduced as a model of integrated care delivery systems. OHTs aim to streamline patient connectivity through the healthcare system and improve outcomes aligned with the Quadruple Aim. When Ontario released a call for health system partners to apply to become an OHT, providers, administrators, and patient/caregiver partners from the Middlesex-London area were quick to respond. We highlight the critical elements and journey of the Middlesex-London Ontario Health Team since its start.

## Introduction

Primary care is seen as the foundation of all health systems and a focus of Canada’s healthcare system.^1,2^ Many of Canada’s richest healthcare policy stories can be recalled with a simple date—1962 (Saskatchewan Doctors Strike); 1966 (*Medical Care Act*); 1984 (*Canada Health Act*); and 2003 (First Ministers Accord on Health Care Renewal). In Ontario, it seems that 2016 and 2019 may earn similar status with the introduction of Bill 41 – Patients First and then Bill 74 (Short titled: *The People’s Health Care Act*, 2019).^3^

Patients First was intended to move the system to more pro-active, primary care-focused, and sustainable integrated care designed around the needs of local populations.^4,5^ Bill 74 then triggered the implementation of the *Connecting Care Act*, 2019, authorizing the Minister of Health and Long-Term Care to create “Ontario Health” (a Crown agency). Bill 74 signaled one of the most significant and structural health reforms towards integrated care ensuring that services would be well coordinated around the broader health needs of the population. Following the lead of health systems around the world, these bills collectively set the stage for integrated care and population health management in Ontario. Ontario Health Teams (OHTs) were introduced as a model of integrated care delivery systems. OHTs were conceptualized to ultimately hold clinical and financial accountability for meeting the primary and secondary care needs of a full attributed population. However, the “how” behind this aim was somewhat elusive and was largely left up to providers, administrators, and citizens across the province to design.

Integrated care and population health management are complex concepts that lack universally accepted standard definitions.^6^ Integrated care is seen as one of the top priorities in health systems globally with variations in how to do it.^7^ However, in some countries, notably the United Kingdom, integration is understood broadly and includes coordination of care across sectors beyond healthcare (for example social care and education).^8^ In Canada, integrated care has taken a narrower focus within traditional healthcare sectors ranging from primary care, mental health, and social services.^9,10^ Ontario’s ambitions to broaden this definition have been evolving for some time. The aim of the OHTs is to streamline the connectivity of patients through the healthcare system and improve outcomes related to the Quadruple Aim.^11^ Population Health Management seeks to shift the overall health and well-being of all members of a populations through data-driven approaches to segmentation and targeted interventions co-designed with each segment.^12,13^ Previous iterations of Ontario’s Regional Health Authorities (RHAs) (notably Local Health Integration Networks, LHINs) have been commonly criticized as lacking the financial independence and collaborative governance to be effective.^14^ In 2019, when Ontario released a call for health system partners to apply to become an OHT, providers, administrators, and patient/caregiver partners from the Middlesex-London area did what many across the province did and got straight to work.

This article highlights eight essential elements that resulted from a collaborative effort by the authors of this article. Although no formal methodology was employed, the authors worked together through a co-design and engagement methodology to identify the key lessons from the Middlesex London Ontario Health Team (MLOHT) transformation journey.^15^ The co-design and engagement methodology was especially beneficial when creating the Equity, Diversity and Inclusion Matrix, the Patient, Client and Care Partner Council, and our decision-making framework. The lessons learned were facilitated through our co-design approach. Our authorship team (which includes researchers, practitioners, and people with lived experience) enabled a range of perspectives across the MLOHT development.^16^

## Coming together (the context in Middlesex)

Middlesex County is located in Southwestern Ontario and home to approximately 500,000 residents.^17^ While the City of London is its most densely populated urban centre, the county’s 3,000 square kilometre area includes diverse rural communities and surrounds three sovereign First Nations (Munsee-Delaware Nation, Chippewas of the Thames First Nation, and Oneida Nation of the Thames). The city is home to Western University, Fanshawe College, two large teaching hospitals (London Health Sciences Centre (LHSC) and St. Joseph’s Health Care London), and a variety of other health service organizations; the county has long been an influential centre for healthcare innovation and education. There has been history of mistrust and fragmentation across the primary care sector in Ontario where providers and organizations have had to compete for and with each other for resources and funding. This is no different in Middlesex-London where health system providers, like so many parts of Ontario, must contend with a legacy of fragmentation and mistrust. At the time Bill 74 was introduced, new leadership in many of these healthcare organizations were coming together, providing space for combining novel ideas with contextual experience to do something different. Early discussions had already begun advocating for collaborative, population-based solutions outside the hospitals and aligning community and organization partners from across the system to achieve it. This gradually created a segue for the development of the Middlesex-London OHT.

In 2019, several key activities were happening in the region. First, local primary care leaders were making grassroots connections to form a Primary Care Alliance (now known as the London Middlesex Primary Care Alliance (LMPCA)), which received support from the Chief Executive Officers (CEOs) of LHSC and St. Joseph’s Health Care London, both of whom were primary care physicians by training. Second, community support services had coordinated around a central intake process to better align their programs, and third, a coalition of local health and community partners had formed the Community Health Collaborative seeking holistic approaches to addressing the social determinants of health. Therefore, when the call for OHT proposals was released in April 2019, Middlesex-London was well-positioned to put forward an application. Building on these opportunities and energized by a secretariat resourced by in-kind contributions from partners, the group was able to quickly engage with regional partners to establish the Western OHT (WOHT). Containing more than 30 signatures from partners, the WOHT’s application was officially approved in July 2020 during a visit to the region by the Deputy Premier and Minister of Health Christine Elliott.

## Early days (committing to a vision)

The OHT approval was an important and solidifying milestone. The relationships and shared principles developed during the application process worked to repair and build trust within the region. The WOHT committed to the principles of stewardship, servant leadership, collaborative governance, decision-making, health equity, integrated care, population health management, and co-design (with patients, clients, caregivers, and providers). There was a need to acknowledge existing fragmentation caused by generations of competing for scarce resources. In parallel, there was a requirement to shift towards system thinking and trust building acknowledging a shared goal and plan for progress. The WOHT started early meetings with reflection and open dialogue, and often included shared education on concepts like health equity, integrated care, and population health management.

One of the first orders of business of the WOHT was the creation of a governance structure. The group agreed on a servant leadership representation model to match the goal of creating a culture of inclusivity and representation within organizations.^18^ The “Coordinating Council” was a representative table of health system senior leaders (chief executive and executive directors), patients and caregivers, and primary care providers from health and social care sectors across the region. Sector representatives (referred to as stewards) on the Coordinating Council demonstrated accountability to the stakeholder sector group on whose behalf they attended, and where possible, linked to existing sector leadership structures (e.g. London Middlesex Primary Care Alliance and Community Support Services Network). The Coordinating Council structure was designed to obtain consensus across all sectors, including those sector groups who were, or felt, traditionally disconnected. Two co-chairs were appointed: one patient/caregiver partner and one primary care administrator. Additionally, an “Operations Team” was created to carry-out the day-to-day work. The Operations Team reports to the Coordinating Council. Some members of the Operations Team are paid from the OHT budget (Ministry of Health funding); other members’ time is provided as in-kind resources from partner organizations. The Operations Team includes individuals with various backgrounds and skills including project management, evaluation and quality improvement, digital health, and communications. Supported by Ministry of Health funding and matched by in-kind resources from hospital and community health partners, the Operations Team helped WOHT to develop and commit to a shared vision, a clear purpose statement, and common values.

An early ministerial requirement for all OHTs was selection of a “year 1 priority population.” Through an evidence-informed, consensus-based decision-making process, partners collectively identified the year 1 priority population as people living with advanced Chronic Obstructive Pulmonary Disease (COPD) and/or Congestive Heart Failure (CHF), who require system navigation/coordination and are at risk for institutionalization. A critical element of this selection process was consensus and evidence that local assets (notably the Best Care Team) could be leveraged within this sub-population to generate improvement in experiences, outcomes, and total cost of care across the system.^19,20^ WOHT leveraged ongoing and established work to support this population as a way to make an even greater impact on the equity-driven Quadruple Aim, specifically health outcomes and patient and provider experiences. It was also felt that this sub-population offered a foundation for change that could demonstrate the principles of integrated care and population health management extending beyond traditional medical care. While prevalence estimates for our region suggested that there are likely over 26,000 people living with COPD and 10,500 with CHF, WOHT chose to start with a target of 2,000 - 3,000 people in the local community with a goal to expand.

## Growing together

In the early days, all OHTs experienced challenges and variability due to lack of centralized standards (e.g. patient/caregiver and care provider compensation), unclear service provision (e.g. Home and Community Care), and digital health. For WOHT, projects were intentionally selected based on their ability to align with and/or inform a provincial population health management strategy. Projects plans were supported by the Operations Team and then vetted and approved by the Coordinating Council, asking questions like “how will this benefit our full attributed population” and “does this align with a vision for equitable care across Ontario; no matter where they live or seek care.” Collaborative decision-making from the Coordinating Council was the key enabler and trust builder for WOHT. The process adopted by the group for consensus decisions supported a safe space for raising concerns and required full transparency and adequate time for dialogue and decision. This included allowing time for dialogue and discussion within a sector before expressing consensus at the OHT Coordinating Council table. Voices were heard from patient partners, clients, and primary care physicians to generate consensus in decision-making. Although time-consuming, this process facilitated trust building within and across sectors. Collaborative decisions around starting new initiatives (such as working to design a compensation strategy) and deferring others were made (participation in the development of centralized and coordinated provincial patient portal strategy). A key facilitator in building trust included providing space for different viewpoints, acknowledging constructive criticism, and not shying away from conflict.

In 2020, the pandemic strained our already challenged health system. In Middlesex-London, the pandemic response galvanized the work of and relationships within the WOHT. Informally, the pandemic was a catalyst to propel the work of the WOHT to near-full capacity in supporting partners in its catchment area. The WOHT supported cultural community COVID-19 vaccine clinics and N95 mask fit testing for community-based staff, and ensured equitable access to COVID-19 remote patient monitoring across the region, including urban and rural geographies. Providers, administrators, and other healthcare workers leaned into the trust and open lines of communication that were built during WOHT development. Established working groups of the WOHT quickly transitioned to focus on pandemic challenges. WOHT development work continued during the pandemic, formalizing the OHT terms of reference and strategic plan, as well as building a Patient, Client and Care Partner Council. This council met monthly to both discuss issues stemming from WOHT coordinating council and develop their own priorities. The Patient, Client and Care Partner Council was empowered to participate in key decisions of the development of WOHT. In parallel, WOHT membership from smaller healthcare and community organizations continued to increase as the coordinating council strived for engagement.

In December 2020, the WOHT received $350,000 in funding from the Ministry for 2020/21 (nearly matching the $368,000 voluntary contribution from partners) and $750,000 for 2021/22 to support development. In early 2021, a WOHT lead was hired. Community-based transfer payments were essential to the success of the WOHT. Support from hospitals (in-kind and monetary) was critical to demonstrate the buy-in and commitment of these large organizations without the expectation of taking the lead. Through co-design and consensus, the OHT adopted a shared purpose statement: “Improving our healthcare experience together – where people are heard, care is connected, and whole health is possible for everyone.” The WOHT also formally adopted an evaluation strategy focused on the “health equity-driven quadruple aim,” a consolidation of the Quadruple Aim (patient outcomes, patient experience, provider experience, and cost of care; endorsed by Ontario Health) and the Quintuple Aim (the addition of health equity) used in other jurisdictions. The evaluation strategy ensures that every piece of work aims to impact one or more elements of the equity-driven Quadruple Aim.

As part of a continued commitment to listening to partners, in February 2022, the WOHT changed its name to the Middlesex-London OHT (MLOHT). This respected partners’ desire to more specifically reference geography and avoid brand confusion with Western University and the provincial Ontario Health regionalization of “Ontario Health West.” Since a name change had been identified as potentially contentious, wide-spread engagement, discussion, and consensus-based decision-making made for a smooth and collaborative process. While strategic decisions like this leveraged collaborative internal processes, OHT work in the community was inclusive and invited collaboration with community groups and stakeholders who had not taken permanent governance seats.

## Lessons learned

The year of 2019 started a wave of healthcare transformation across Ontario, but it will be some time before we truly know the impact of this reform. To date, 54 OHTs are at various stages of development across the province. In Middlesex-London, we are confident that the movement to an OHT model has led to a stronger health system working towards a shared purpose of improved healthcare for our community owing to our commitment to co-design, population health management leadership, and primary care engagement and partnership. This has allowed us to be provincial leaders in health information interconnectedness, co-design, and care pathway implementation (e.g. Quality Based Procedure (QBP) for CHF program). We continue to strive for a balance between action-oriented projects and longer-term initiatives like attachment to primary care and centralized referral management.

We believe our success can be boiled down to eight essential elements. These elements and associated descriptions are summarized in Table 1.

**Table 1.** WOHT eight essential elements.

Essential elements	Description
(1) Shared dedication to a vision, purpose statement, and common values	There is a community-wide, shared dedication to the vision and purpose of the WOHT.
(2) Created a culture of inclusive representation	Across all of the WOHT work, special attention is given to ensuring that our Equity, Diversity, and Inclusion Matrix is being referenced—Are we being mindful of whose voices are at the table? Are we making decisions that will benefit the entire community?
(3) Empowered and supported Patient, Client and Care Partner (PCCP) Council	The PCCP Council reports directly into the Coordinating Council and has autonomy to make decisions about their own work plan, as well as provide input into Coordinating Council decision-making. The Council has support from two Operations Team members as needed.
(4) Developed a strong Operations Team (in part, due to in-kind support from partner organizations)	The Operations Team is made up of individuals with a variety of skills including clinical leadership, project management, communications, and quality improvement. Supported by Ministry of Health funding and matched by in-kind resources from hospital and community health partners, the Operations Team helped WOHT to develop and commit to a shared vision, a clear purpose statement, and common values.
(5) Community-based transfer payments with tremendous “leading from behind” support from hospitals	We intentionally selected a community-based health service provider, Thames Valley Family Health Team, to be our Transfer Payment Recipient for our OHT Operational funds. However, we receive tremendous support from our hospital partners (e.g. in-kind human resources dedicated to population health management, community partnerships, co-design).
(6) Commitment to co-design and meaningful engagement with patients, caregivers, providers, and administrators	Co-design and engagement approaches have been embedded across all levels of the WOHT structure. Patient and caregiver partner representatives are included in every project that the WOHT undertakes. Special efforts continue to be made to gather input and feedback from individuals with diverse experiences.
(7) Adopted a consensus-based decision-making framework	We have clearly documented our shared understanding of who/which groups are empowered to make what level of decisions and our process for doing so using a decision tool and level of consensus matrix (1-6 scale).
(8) Grassroots leadership from primary care providers (through LMPCA)	The LMPCA provided support to the WOHT with the help in the application and dedication to a sector approach in primary care.

All our work is grounded in health equity, quality improvement principles, population health, and co-design approaches. We are committed to building respectful, trusting, reciprocal relationships with local First Nations and the broader local Indigenous community, formal and informal collaboration with neighbouring OHTs (including hiring of a shared Indigenous Health Improvement Facilitator role), as well as partnering with researchers. Through all of this, we aim to position our work in service to the overall OHT strategy, policies, and processes that hold us accountable to each other and our community.

## Looking forward

As we look ahead to the future of the MLOHT and the future of integrated care, we must be mindful of where we came from and what is needed for success long term. Our shared vision for equitable access to better, more integrated care will continue to motivate those engaged with the MLOHT. The MLOHT working groups, coordinating council, patient/client care partner council, and Operations Team continue to inspire hope in the community that positive change is within reach. We hope that others can learn from our journey, and we remain committed to learning from others as we work together towards a future of stronger, more integrated care for everyone.

The limitations of our article include its primary focus on lessons learned from integrated care work in the London-Middlesex region, which may not necessarily be applicable to other regions. We are aware that our context is a huge factor in our journey. We know the COVID-19 pandemic caused setbacks for many people in many sectors; however, MLOHT was able to galvanize existing momentum to take action and grow during the pandemic crisis. Additionally, while our authors represent a diverse range of perspectives, we know we may not have captured every voice in our discussion of the MLOHT's success. We are committed to continuing to engage our community, and in the future, we will use more systematic methods (such as interviews or focus groups) to gather a more comprehensive range of perspectives on the MLOHT's journey.

## References

[1] Glazier R . Balancing equity issues in health systems: perspectives of primary healthcare. Healthc Pap. 2007;8(sp):35-45. doi:10.12927/hcpap.2007.19218 19096264

[2] Glazier RH . Our role in making the Canadian health care system one of the world's best: how family medicine and primary care can transform-and bring the rest of the system with us. Can Fam Physician. 2023;69(1):11-16. doi:10.46747/cfp.690111 36693751 PMC9873296

[3] E-laws . Ontario.ca. https://www.ontario.ca/laws/view. Published October 10, 2019. Accessed March 7, 2023.

[4] Lavis JN . Hamilton, ON: McMaster Health Forum; 2019:1-6. https://www.mcmasterforum.org/docs/default-source/rise-docs/rise-briefs/rb1_oht-building-blocks.pdf?sfvrsn=71b154d5_27.

[5] Gordon D McKay S Marchildon G Bhatia RS Shaw J . Collaborative governance for integrated care: insights from a policy stakeholder dialogue. Int J Integr Care. 2020;20(1):3-11. doi:10.5334/ijic.4684 PMC7019202 32089655

[6] Goodwin N. Understanding integrated care. Int J Integr Care. 2016;16(4). doi:10.5334/ijic.2530 PMC5354214 28316546

[7] Mounier-Jack S Mayhew SH Mays N . Integrated care: learning between high-income, and low- and middle-income country health systems. Health Policy Plan. 2017;32(suppl_4):iv6-iv12. doi:10.1093/heapol/czx039 29194541 PMC5886259

[8] Charles A . Integrated care systems explained. The King's Fund. https://www.kingsfund.org.uk/publications/integrated-care-systems-explained. Published August 19, 2022. Accessed March 7, 2023.

[9] Ministry of Health Ontario . Become an Ontario health team. Ontario Health Teams. https://health.gov.on.ca/en/pro/programs/connectedcare/oht/. Published 2023. Accessed March 8, 2023.

[10] Leatt P Pink G Guerriere M . Towards a Canadian model of integrated healthcare. Healthc Pap. 2000;1(2):13-35. doi:10.12927/hcpap.17216 12811063

[11] Ontario Medical Association . Ontario health teams: a new integrated health-care delivery system. Ontario Health Teams. https://www.oma.org/advocacy/ontario-health-teams/. Accessed March 7, 2023.

[12] Cronin S Tessier L James KA Wodchis WP. Approaches to population health management: informing Ontario’s health system transformation. Health System Performance Network, 2021:1-28.

[13] Farmanova E Baker GR Cohen D . Combining integration of care and a population health approach: a scoping review of redesign strategies and interventions, and their impact. Int J Integr Care. 2019;19(2):5. Published 2019 Apr 11. doi:10.5334/ijic.4197 PMC6460499 30992698

[14] Uchimura LY Viana AL Marchildon GP . Managers and clinicians: perceptions of the impact of regionalization in two regions in Canada. Healthc Manage Forum. 2019;32(3):163-166. doi:10.1177/0840470418817913 30947552

[15] Donetto S Pierri P Tsianakas V Robert G . Experience-based co-design and healthcare improvement: realizing participatory design in the public sector. The Design Journal. 2015;18(2):227–248. doi:10.2752/175630615x14212498964312

[16] Dalglish SL Khalid H McMahon SA . Document analysis in health policy research: the read approach. Health Policy Plan. 2021;35(10):1424-1431. doi:10.1093/heapol/czaa064 33175972 PMC7886435

[17] Government of Canada . Middlesex county census profile, 2021. Ontario. https://www12.statcan.gc.ca/census-recensement/2021/dp-pd/prof/details/page.cfm?Lang=E&SearchText=Middlesex&DGUIDlist=2021A00033539&GENDERlist=1%2C2%2C3&STATISTIClist=1&HEADERlist=0. Published February 1, 2023. Accessed March 7, 2023.

[18] Canavesi A Minelli E . Servant leadership: a systematic literature review and network analysis. Employ Respons Rights J. 2021;34(3):267-289. doi:10.1007/s10672-021-09381-3 PMC8476984 40477405

[19] Hussey AJ Sibbald SL Ferrone M , et al. Confronting complexity and supporting transformation through health systems mapping: a case study. BMC Health Serv Res. 2021;21(1). doi:10.1186/s12913-021-07168-8 PMC8540206 34688279

[20] Sibbald SL Misra V daSilva M Licskai C . A framework to support the progressive implementation of integrated team-based care for the management of COPD: a collective case study. BMC Health Serv Res. 2022;22(1). doi:10.1186/s12913-022-07785-x PMC8966237 35354444

